# Acupressure for the Arrest of Hemorrhage

**Published:** 1860-10

**Authors:** 


### ARTICLE XVII.

### *Acupressure for the Arrest of Hemorrhage.*

At a late meeting (April 24th, 1860,) of the Royal Medical and Chirurg. Society, Mr. Syme, of Edinburg, in reply to a question by Mr. Henry Thompson, said that acupressure was not, in his opinion, calculated to improve the practice of surgery. In the first place, he did not think the objections alleged against the ligature were fully justified ; in the second place, if the ligatures were objectionable, they had a better substitute for it in torsion than in the needle process ; thirdly, he thought the needle process was hardly practicable, and in some cases not practicable at all. It had been said by the proposer of the method that the ligature would occasion gangrene. That simply showed that the individual who proposed it did not know the meaning of surgical language. As to ligature causing irritation, that assertion was not true. He had repeatedly tied the femoral, and left the wound to heal by first intention, there being only a few drops of matter, and in one case not a drop, showing that the ligature was not the cause of irritation. He had given up the practice of cutting away one thread, always preserved both ; and he regarded ligatures rather as useful assistants than as obstacles. After amputation, the great impediment to union by the first intention was the presence of blood, which coagulated, and led to the formation of abscess. The ligatures prevented this, made way for the discharge, and did good rather than harm. But if ligatures were sometimes objectionable, the process of torsion was a convenient substitute, the success of which he had repeatedly seen. As to the acupressure, it could very rarely be employed ; and his only surprise was that any practical surgeon should have given it two thoughts. It was a tub thrown out to amuse the whale, more especially to feed the whale, and would never have been heard of had it not been brought under the notice of the profession by a medical journal published in London, understood to be under the control of the proposer.

Mr. Spencer Wells said he should not notice the personalities with which Mr. Syme concluded his speech; but with regard to acupressure he thought it but just to say that he had employed that method in a case of Pirogoff's amputation at the ankle-joint, in which the needle was fairly pitted against the ligature. The anterior tibial artery was compressed by the needle, and a plantar branch of the posterior tibial was tied by a ligature. The superiority of the needle was most marked. In forty-eight hours Mr. Adams, whose case it was, removed the needle, and there was no more trouble about it. The ligature remained some five or six days afterwards, setting up suppuration in its track, and keeping open the wound in the manner which Mr. Syme appeared to think so favorable (the patient thought otherwise,) but which certainly delayed the cure. The case would have been better treated if it had been easy to compress the posterior tibial artery in the same manner as the anterior tibial, if he had not to learn the A B C of acupressure, as all must do. There might be many cases in which the needle is not applicable; but he believed if he had known then what he had learned since, he might have been able to compress the plantar artery as easily as the other. So far from the introduction of acupressure exhibiting any want of surgical knowledge on the part of the gentlemen who proposed it, he believed it to be one of the many gifts, including the introduction of chloroform, for which surgery is indebted to that great man. Mr. Wells further expressed his conviction that acupressure will prove a most useful means of suppressing hemorrhage, and he had learned its utility in compressing varicose veins. He believed, also, that it will hereafter supersede the ligature in the treatment of aneurism. With regard to the effect of the ligature upon the ends of divided arteries, every surgeon knew that the part of the artery beyond the ligature must be killed by it, and that a piece of sloughy tissue cannot do any good when confined amid the living tissues of the body, however useful Mr. Syme might consider it to be.

*Med. Times and Gazette, May* 5, 1860.

